# Family Influences on Antiretroviral Therapy Adherence in Youth with HIV in the United States: A Systematic Review

**DOI:** 10.1007/s10461-025-04721-y

**Published:** 2025-04-28

**Authors:** Violeta J. Rodriguez, Miranda Gessert, Arianna Guerra

**Affiliations:** Department of Psychology, University of Illinois Urbana Champaign, Champaign, USA

**Keywords:** HIV-positive youth, ART adherence, Family-level Influences, Family-based Interventions, Barriers

## Abstract

HIV poses a significant issue in the United States and understanding how HIV-positive populations adhere to treatments, specifically with antiretroviral therapy (ART), is vital for researchers to find approaches to improve medication adherence. This systematic review aims to explore barriers that children, adolescents, and young people (ages < 25) in the United States may face by exploring family interventions and factors influencing ART adherence. This study conducted a literature search using various databases to retrieve studies within the United States. A total of 36 studies identified family factors (*n = 29*) and various interventions (*n = 7*). Across both categories, studies indicated the critical role of family involvement in adherence outcomes. The review also identified significant barriers to ART adherence, including complex medication regimens, HIV-related stigma, and psychosocial stressors. Due to the diverse barriers, policymakers and healthcare providers should focus on a holistic approach to support ART adherence in HIV-positive youth.

## Introduction

The HIV epidemic continues to be a significant public health challenge in the United States, particularly among children, adolescents, and young people. In the US, adolescents and young adults (ages 13–25) account for 20% of new annual cases in 2022 [1]. Despite advancements in medical treatments, including antiretroviral therapy (ART), adherence to these therapies remains a critical issue. Effective ART adherence is essential for suppressing HIV viral load, improving health, and reducing the transmission of HIV. However, maintaining consistent adherence to ART can be particularly challenging for younger populations due to various psychosocial, developmental, and environmental factors. For example, among adolescents and young adults with HIV, only 45% of adolescents and young adults are engaged in HIV care, and a mere 37% achieve viral suppression, the lowest rates among all age groups [2]. The transition from pediatric to adult care exacerbates these challenges, with ~ 45% of youth with HIV lost during this critical period [3].

Given the multitude of barriers faced by youth with HIV throughout the HIV care cascade, mathematical models predict stark reductions in life expectancy in this population. Youth with perinatally acquired HIV (i.e., HIV transmitted from mother to child during pregnancy, childbirth, or breastfeeding) face life expectancy losses of 10 years for male and 12 years for female youth with HIV, while those with non-perinatally acquired HIV (i.e., HIV acquired through behaviors such as shared needles, condomless [anal] sex, or other high-risk behaviors) are expected to experience even greater losses: 15 years for male and 20 years for female youth with HIV [4]. In an ideal HIV care scenario, these life expectancy losses could be significantly mitigated to 0.5 years for male, 0.6 years for female youth with perinatally acquired HIV, 6 years for male, and 10 years for female youth with non-perinatally acquired HIV [4].

While youth with HIV face significant risks related to HIV and intersecting identities that compound these risks (e.g., sexual and gender identities, racial and ethnic identities), they demonstrate remarkable resilience. In youth with HIV, supportive family functioning predicts resilience, which in turn predicts positive community and peer relations, highlighting youth with HIV’s strengths [5]. Qualitative approaches have also pointed to similar interpersonal sources of resilience in youth with HIV and sexual and gender minorities of color [6]. For sexual and gender minorities with HIV, enlisting social support from families and peers is critical to enhance their resilience and well-being [6]. Sexual and gender minorities with HIV report that family members often assist in coping with life stressors, providing constructive perspectives on both day-to-day decisions and longer-term life goals [6]. For instance, they describe how family reassurance and affirmation regarding life directions significantly motivated and supported their mental health [6]. Therefore, the family, as a primary source of support, may play a significant role in fostering resilience by providing emotional and practical support, which is crucial for the healthy development and adaptation of youth HIV in the face of adversity. Therefore, HIV guidelines suggest that addressing health disparities requires the consideration of family-level influences on ART adherence, particularly for marginalized populations [7, 8]. Additionally, family-level factors, such as parental support, family communication, and overall family environment, may be integral to the adherence behaviors of youth with HIV.

Despite the potential benefits, the impact of family-based interventions and family-level factors on ART adherence among youth in the United States is not fully understood, despite their frequent use and success in low and middle-income countries [9]. In the few studies examining family-level factors, it has been shown that in youth with HIV, supportive family functioning predicts resilience, which in turn predicts positive community and peer relations [5]. Thus, in this systematic review, we aim to synthesize the existing literature on the impact of family-based interventions and family-level influences on ART adherence in youth with HIV in the United States. By examining the effectiveness of these interventions and identifying key family-related factors, this review seeks to provide a comprehensive understanding of how family dynamics can be leveraged to improve ART adherence in this vulnerable population in the United States. The findings of this review will inform future research and guide the development of targeted interventions to enhance ART adherence among young people, ultimately contributing to better health outcomes and quality of life for this population. Specifically, our systematic review will address three research questions. First, what is the impact of family-based interventions on ART adherence in youth with HIV in the United States? Second, what family-level factors influence ART adherence in this population? Third, how do family dynamics mediate or moderate ART adherence behaviors in youth with HIV?

Given the existing literature on the role of family dynamics in health behaviors, we make three major hypotheses [6, 10]. First, we expect to find that family-based interventions significantly improve ART adherence in youth with HIV. We anticipate that a supportive family environment characterized by parental support, effective family communication, and overall positive family functioning is associated with better ART adherence. In addition, we hypothesized that family dynamics, such as parental involvement, communication patterns, and emotional support, moderate ART adherence behaviors in youth with HIV. Specifically, we expect that youth who report higher levels of family support, open communication with caregivers, and positive family functioning will exhibit better adherence to ART. Conversely, families with poor communication, low levels of support, or conflict may contribute to lower adherence behaviors in youth with HIV. Based on previous research with sexual and gender minority youth in primary care [6, 10], we also sought to explore whether family-based influences may be particularly salient for marginalized populations, such as sexual, gender, racial, and ethnic minority youth, due to the additional social and systemic challenges they face.

## Expectations Regarding Sources

We expect this literature to be limited and heterogeneous. As such, we expect to encounter a variety of study designs, including cross-sectional, observational, intervention studies, randomized controlled trials, quasi-experimental, cohort studies, case-control, mixed methods, and qualitative studies. We anticipate a wide range of measurement tools used to assess ART adherence and family-level influences, including self-report surveys, objective measures (e.g., pill counts, electronic monitoring), and qualitative interviews. The studies are likely to reflect diverse geographical locations within the United States and varying demographic characteristics (e.g., age, gender, race, ethnicity, sexual identity). We recognize that healthcare disparities and access to healthcare resources can influence the effectiveness of family-based interventions. The social stigma and discrimination associated with HIV and minority statuses (e.g., racial/ethnic, sexual orientation) may impact ART adherence and the efficacy of family-based interventions. These contextual factors will be considered when interpreting findings.

### Methods

#### Reporting and Registration

Before study implementation, we developed a review protocol registered on OSF.io (https://osf.io/nxf8r/?view_only=d04cfb38fa44486fadb4be34306e3767). The review was conducted following the guidelines for scoping reviews described by the JBI Manual for Evidence Synthesis and Preferred Reporting Items for Systematic Reviews and Meta-Analyses (PRISMA) [11].

## Search Strategy

Three search strings were combined to identify studies that assessed family interventions or factors relating to ART adherence in children, adolescents, and young people. With each concept in our search strategy, we included the Medical Subject Headings terms relative to our study as outlined in the National Library of Medicine’s database. After refining our search, the final search strategy was (“United States” OR USA OR U.S. OR “United States of America”) AND (child OR children OR adolescent* OR teens* OR youth OR “young people”) AND (“HIV infection*” OR AIDS OR “Acquired Immunodeficiency Syndrome”) AND (Family OR families OR “Family therapy” OR “Family-based intervention*” OR “Family support” OR “Family counseling” OR “family counselling” OR “Family involvement” OR “Family engagement” OR “Family Functioning” OR “Family Therapy”) AND (“highly active antiretroviral therapy” OR “ART Adherence” OR “antiretroviral therapy adherence” OR “combination antiretroviral therap*” OR “ART compliance” OR “combination antiretroviral compliance” OR (“medication adherence” OR “medication compliance”) AND (“anti-retroviral agents” OR “anti-HIV agents” OR “HIV infection*” OR AIDS OR “acquired immunodeficiency syndrome”)). The search strategy was conducted on 20–21 June 2024 on the EBSCOhost platform of the following databases: Academic Search Premier, CINAHL Plus, MEDLINE, APAPsycArticles, LGBTQ + Source, and PsycINFO.

## Selection Criteria

Our review included cross-sectional data from observational studies, qualitative or mixed studies, survey research, intervention studies, or randomized controlled trials, as these research designs were deemed to be the most appropriate for synthesizing the literature on the influence of family interventions and influences on ART adherence in this population. All designs constituting original, peer-reviewed research were considered for the qualitative synthesis if they examined family interventions and factors on ART adherence. ART adherence was defined as the use of any measure of ART adherence, whether via self-report or objective measures, whether self-administered or administered by a researcher. Similarly, family influences were defined broadly, as studies were included if they included at least one family-related measure or consideration (e.g., questions related to family influences in qualitative designs). Studies were not excluded based on language. Of those papers reporting studies sharing a common data source (e.g. if multiple papers reported on data from the same survey study), we included only one of those publications in order not to artificially inflate our sample size. Studies (1) not focused on the United States population; (2) not involving children, adolescents, or young people (defined as those younger than 25); and (3) not addressing ART adherence, or non-primary or secondary sources (e.g., editorial, opinions) were not included. The resulting articles were imported into Endnote v.9 and de-duplicated. The Endnote library was imported into Covidence, an online systematic review management software, and de-duplicated again.

## Type of Sources

This review included peer-reviewed publications that explored family interventions or influences relating to ART adherence in youth with HIV, defined as children, adolescents, and young people, including experimental, observational studies (i.e., quasi-experimental, cohort studies, case-control, cross-sectional, case series), mixed methods, or qualitative studies). Secondary analyses, such as systematic reviews and meta-analyses, were included if they met the inclusion criteria. This review will not include grey literature due to the difficulty in retrieving and extracting the data, but also in evaluating its credibility due to the absence of standardized quality indicators or peer review processes.

### Study Selection

Following the removal of duplicates and import into Covidence, the initial citations were screened against inclusion/exclusion criteria by title and abstract. Review and commentary articles were set aside for a manual search of their included studies. The remaining citations were screened by full-text perusal and those found to adhere to all selection criteria were selected for review. The reference lists of the selected studies were manually searched for additional articles. A full review of all eligible citations was conducted by both authors. All eligible studies were also reviewed at each stage of screening by both authors, as were any studies under question, and discrepancies were addressed through discussion until consensus was reached. The justification for excluding articles following screening the full text was recorded.

Given the absence of prior reviews on this topic and in this population in the United States, no date limitations were set. The reference and bibliographic lists of all studies included in the review were searched to minimize the likelihood of missed citations. In addition, any systematic reviews identified during the literature search that presented data on topics related to the primary research aim were also searched manually.

## Data Extraction and Risk of Bias Assessment

Papers selected for review were re-read thoroughly with data extracted into pre-prepared tables outlining study characteristics, outcomes of interest (ART adherence and how it was measured, and independent variables of interest (family influences and how they were measured), even if family influences were measured as an indirect influence (e.g., as interaction effects or mediation effects). Further to this, papers were read in the full text once more to identify other notable findings relating to the topic of interest, which were categorized and tabulated heuristically. The template for data extraction was drafted during the pre-review protocol development phase with agreement from all authors. Data extraction was conducted by one reviewer (MG) with a selected sample (10% alongside any data under question) checked by another reviewer (VJR). Any discrepancies were addressed through discussion until a consensus was reached.

## Data Analysis and Presentation

The results were presented in a narrative summary, along with tables and charts. Gaps in the evidence were identified at this stage. PRISMA was completed along with the presentation of results to ensure each element was covered within the review.

## Results

### Study Design

**Fig. 1:**
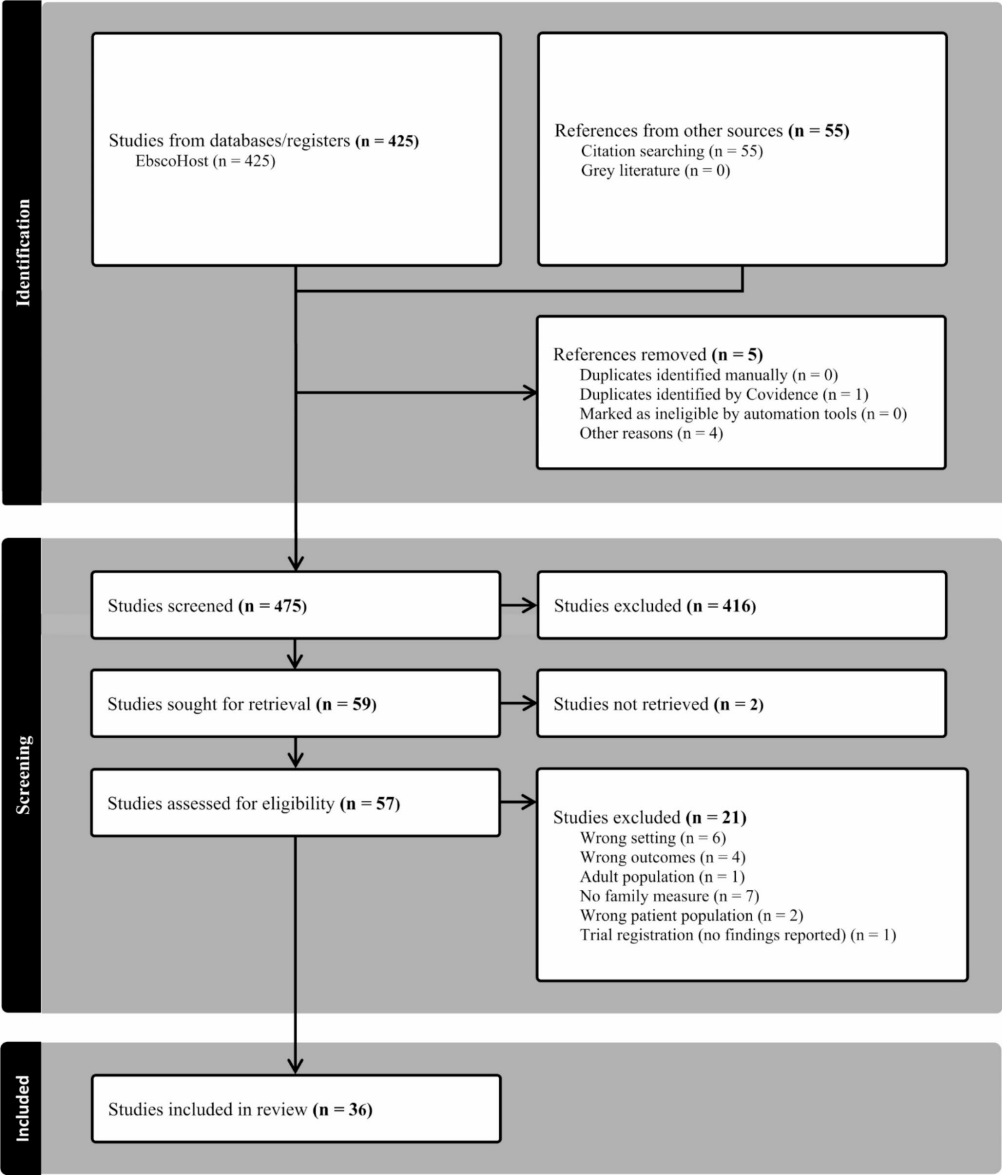
PRISMA flow diagram of study selection for systematic review on family influences and ART adherence

A total of 36 studies were included in this review. Of the 36 studies, 11 utilized a cross-sectional study design, and 9 used a qualitative approach, which included descriptive analysis, a qualitative systematic review, and a literature review. Other methods included longitudinal (*n* = 7) [12–18], case report (*n* = 2) [19, 20], retrospective chart review (*n* = 2) [21, 22], non-blinded randomized controlled trial (*n* = 1) [23], mixed methods (*n* = 1) [24], and cohort studies (*n* = 1) [25]. These are summarized in Fig. 1.

### Study Demographics

All studies were conducted in the United States. As described in Table 1, of these, 23 studies focused on HIV-infected children/adolescents as their target population, while 8 studies [14, 18, 26–31] studied perinatally infected children and their families. The other 6 studies did not account for the HIV-positive child; rather 3 studies focused on the caregiver, 1 study focused on the medical provider, and 2 studies synthesized a collection of HIV studies. The median sample size was 307 (range 1–5733) with participants’ ages ranging from 0 [32] to 25 years [33]. A total of 22 studies used sex, as opposed to gender, to measure the population’s identity, while 8 studies did not measure gender or sex. Overall, studies were evenly split among males and females. Only 2 studies measured sexual orientation in their sample [21, 34]. Three studies examining sex associations with adherence found no significant or consistent associations between sex or gender and adherence [24, 25, 30].

**Table 1:** Study characteristics and participant demographics

Author(s) (Year)	Study Design	Population	Sample Size	Age Range	Sexual Orientation	Gender	Sex	Race	Ethnicity
Allison et al. [12]	Longitudinal	Children infected with HIV prescribed ARV treatment at baseline	126	2.9–15.1 years	Not measured	Measured as sex	61% female	76% non-Hispanic African American, 16% Hispanic, 6% non-Hispanic Caucasian	Measured with race
Berrien et al. [23]	Non-blinded randomized controlled trial	HIV-positive patients enrolled in Connecticut Children’s Medical Center’s (CCMC’s) pediatric and youth HIV program	37	1.5–20 years	Not measured	Measured as sex	Intervention group 55% female, control group 45% female	Intervention: 50% Hispanic, 35% African American, 15% Caucasian; Control: 64% Hispanic, 29% African American, 7% Caucasian	Measured with race
Brackis-Cott et al. [47]	Qualitative	Medical providers working with perinatally HIV-infected children	10	Caring for children approximately 6–13 years	Not measured	Measured as sex	80% women	70% white, 30% African American	Not measured
Buchanan et al. [13]	Longitudinal	Perinatally infected children and adolescents	120 dyads	8–18 years	Not measured	54% boys	Measured as gender	55% African American	28% Hispanic
Byrne et al. [14]	Qualitative, longitudinal	HIV-seropositive children receiving ARV meds for HIV and their families	42	4 months − 18 years	Not measured	Same as sex	52% female	34% African American, 39% Latino, 5% White, 10% biracial, 12% other	Measured with race
Childs & Cincotta [32]	Qualitative	HIV-infected children	Not measured	0–19 years	Not measured	Not measured	Not measured	Not measured	Not measured
Cunningham et al. [16]	Case report	Poorly adherent, perinatally infected adolescents	1	12 years	Not measured	Boy	Measured as gender	African American	Not measured
Davey et al. [21]	Retrospective chart review	Urban minority youth with HIV	50	14–24 years	65% heterosexual, 35% MSM	Measured as sex	57% male, 43% female	92% African American, 8% Latino/a	Measured as either African American or Latino/a
Dodds et al. [33]	Descriptive analysis	HIV-infected young women and adolescents	21	20–25 years	Not measured	Same as sex	All female	52% African American, 28.5% Caribbean origin, 14% Hispanic, 1 White non-Hispanic	Measured with race
Dolezal et al. [26]	Cross-sectional	HIV-infected child and primary caregiver was either a parent, relative, or adoptive	48 adult-child dyads	3–13 years	Not measured	Same as sex	56% female	73% African American/Back, 25% Latino/Hispanic, 2% mixed	Measured with race
Ellis et al. [22]	Retrospective chart review	Children perinatally infected with HIV	19	20 months- 16 years	Not measured	Measured as sex	62% male, 38% female	84% African American, 11% Caucasian	Not measured
Fair et al. [15]	Longitudinal, mixed methods case study	HIV-positive pediatric patients	15	10–21 years	Not measured	Measured as sex	64.9% female, 35.1% male	62.1% African American, 29.7% African, 8.1% Hispanic	Measured with race
Fields et al. [34]	Qualitative	Behaviorally infected and perinatally infected youth	30	14–24 years	P: 11% gay/bisexual/other, 89% straight; B: 92% gay/bisexual/other, 8% Straight	P: 33% male, 67% female; B: 83% male, 8.5% transgender male, 8.5% transgender female	Only gender was measured	P: 55% Black, 6% White, 39% Other; B: 25% Black, 42% White, 33% Other	P: 44% Latino; B: 25% Latino
Gray et al. [35]	Case series intervention	HIV-positive adolescents with poor adherence	4	13–17 years	Not measured	Measured as sex	75% female, 25% male	75% Black, 25% White	25% Hispanic
Kacanek et al. [16]	Longitudinal	Perinatally acquired HIV youth	381	8–22 years	Not measured	Same as sex	53% female	74% Black	23% Latino
Katko et al. [21]	Cross-sectional	HIV-infected children and their caregivers	24 children and 35 caregivers	1.5–19.9 years	Not measured	About 37% male	Measured as gender	Not measured	Not measured
Letourneau et al. [20]	Case study	Young woman diagnosed with perinatally acquired HIV	1	17 years	Not measured	Woman	Female	White	Not measured
Lyon et al. [28]	Intervention	HIV-positive youths and their family members	23	15–22 years	Not measured	34.8% male, 60.9% female, 4.3% transgender MTF	Only gender was measured	100% African American	Not measured
Malee et al. [36]	Cross-sectional	Children with perinatally acquired HIV and on ART	1134	3–17 years	Not measured	Measured as sex	52% female	13% White, 61% African American, 24% Latino	Measured with race
Marhefka et al. [29]	Mixed-methods, cross-sectional	Perinatally HIV-infected children and their caregivers	51	2–13 years	Not measured	47.1% female	Measured as gender	82.4% African American, 9.8% Caucasian, 7.9% other	Not measured
Marhefka et al. [46]	Cross-sectional, descriptive	Caregivers of perinatally HIV-infected children under 13 years old	54	1–13 years	Not measured	Measured as sex	52% male	83% African American	Not measured
Martin et al. [17]	Longitudinal/cohort	Children who have vertically acquired HIV disease and have been on a HAART regimen that included a protease inhibitor	24	8–18 years	Not measured	Measured as sex	54% male, 46% female	46% Black, 42% White, 4% Hispanic, 8% Native American	Measured with race
Mellins et al. [37]	Cross-sectional	Perinatally HIV-infected children	77	3–13 years	Not measured	Same as sex	54% female	64% African American	30% Latino
Mellins et al. [30]	Cross-sectional	Perinatally HIV-infected children and their caregivers	75	3–13 years	Not measured	Measured as sex	56% female	69% Black, 25% Latino	Measured with race
Naar-King et al. [31]	Cross-sectional	Children perinatally infected with HIV and their caregivers	43	2–17 years	Not measured	Measured as sex	51% female	79% African American, 7% European American, 5% Hispanic, 9% mixed ethnicity	Measured with race
Naar-King et al. [38]	Cross-sectional	Youth perinatally infected with HIV	123	8–18 years	Not measured	Not measured	Not measured	58% African American, 28% Hispanic	Measured with race
Nicholson et al. [44]	Cross-sectional, descriptive	Children with perinatally acquired HIV	75	3–13 years	Not measured	Same as sex	82% female	61% African American, 30% Latino	Measured with race
Rao et al. [39]	Qualitative	Patients receiving treatment for HIV in adolescent and young adult clinic	25	17–25 years	Not measured	Measured as sex	52% male, 48% female	88% African American, 8% mixed, 4% Latino	Measured with race
Reddington et al. [18]	Longitudinal, mixed methods	HIV-infected children and their caregivers	169 children and (90 parents)	1.1–14 years	Not measured	Not measured	Not measured	Not measured	Not measured
Reisner et al. [24]	Mixed methods	HIV-infected youth	5733 (21 studies)	13–24 years	Not measured	Same as sex	Complied studies (some had male/female, others just adolescents)	Not measured	Not measured
Simoni et al. [45]	Qualitative systematic review	work from January 1996 to December 2005 that included the 3 terms “HIV/AIDS,” “pediatric,” and “compliance/adherence”	stated as ~ 50	3 months- 24 years	Not measured	Not measured	Not measured	Not measured	Not measured
Steele & Grauer [40]	Literature review	Studies were included if they reported adherence as an independent outcome, involved children and/or adolescents with HIV/AIDS, and clearly described the adherence assessment method.	13	differs between studies; average ages for each study given	Not measured	Not measured	Not measured	Not measured	Not measured
Steele et al. [40]	Cohort	Clinic patients who were diagnosed with HIV infection and who were prescribed at least 1 antiretroviral	30	0.9–11.5 years	Not measured	Measured as sex	40% female	All except one participant was African American	Not measured
Thrul et al. [41]	Qualitative	HIV care providers of AYA-HIV aged 15 to 24 years	9	not measured	Not measured	Not measured	Not measured	Not measured	Not measured
Williams et al. [42]	Cross-sectional	Children and adolescents with perinatally acquired HIV infection	2088	3–18 years	Not measured	Measured as sex	52% female	16% White/others, 59% Black, 25% Hispanic	Measured with race
Wrubel et al. [43]	Qualitative	Maternal caregivers of children with HIV	71	1–18 years (child)	Not measured	Not measured	Not measured	45% African American, 27% White, 21% Latina, 7% other (caregiver) - child race not measured	Measured with race

When considering race and ethnicity, 21 studies included race and ethnicity in the sample, while 8 studies only measured race, and 7 studies did not measure racial or ethnic identities. Of the 29 studies, 26 had samples of primarily minority racial and ethnic groups, with 24 studies having a predominantly Black/African American demographic, while 2 had predominantly Hispanic/Latino participants. A total of six studies examined differences in relation to racial and ethnic identities. Four of these studies found either no association, or inconsistent associations between either race or ethnicity and adherence-related factors [12, 24, 30, 44]. One study found increased nonadherence among African American and Latino participants [36], while another found that status disclosure to others was more likely among African American than Latino participants [37]. One study examined race and ethnicity in relation to both adherence and viral load and found that although any associations with viral load were statistically insignificant, identifying as Black was associated with nonadherence [16].

### Intervention Types

Of the 36 studies reviewed, 7 were intervention-based. As noted in Table 2, these studies all focused on home and family-based interventions [15, 19, 20, 22, 23, 28, 35]. A total of *n* = 3 of these studies specifically implemented multisystemic therapy (MST), a strategy in which psychotherapy is provided based on an assessment of what barriers are preventing successful outcomes for patients [19, 20, 22]. Additionally, 1 study was implemented in a focus group setting [28].

**Table 2:** Intervention or IV and outcome measures

Authors (Year)	Intervention?	Family-level factor	Outcome measure (ART adherence)	Measurement tools (Family)	Key findings
Allison et al. [12]	Longitudinal study that examined the rate of and risk factors for perinatal HIV transmission in 4 US locations (Atlanta, GA; Baltimore, MD, Newark, NJ; New York, NY) following HIV-infected children from Oct 1985 to Sep 1999.	Caregiver knowledge of prescribed doses, parental recall of missed doses, and caregiver difficulty administering medication	Viral load and missed doses reported by caregiver	Modified Pediatric AIDS Clinical Trials Group (PACTG) adherence interview	Caregiver knowledge of prescribed doses (*p* =.01) and caregiver difficulty administering medication (*p* =.03) were associated with higher viral load. Parental recall of missed doses was not associated with viral load.
Berrien et al. [23]	Home-based nursing intervention	Nurses helped educate caregivers and children about HIV and medication, along with giving them tools and strategies to improve adherence	Pharmacy refill records, self-reported adherence, viral load, and CD4 count	Pre- and post-study questionnaires	The intervention improved HIV and medication knowledge (*p* =.02), and pharmacy refill frequency (*p* =.002) among the intervention group. Self-reported adherence improved, but it was not statistically significant (*p* =.07).
Brackis-Cott et al. [47]	Qualitative interview	HIV primary care providers’ relationships with patients and adherence communication strategies	Antiretroviral treatment decisions, strategies used for engaging families in ongoing HIV/AIDS treatment services, working with difficult caregivers, and disclosure issues	Qualitative interviews with healthcare providers	In general, the providers believed that there were not enough available options for families needing treatment and that this caused increased struggles with adherence.
Buchanan et al. [13]	Longitudinal study	Child- and caregiver-reported barriers to adherence	Pill count and child- and caregiver-reported adherence	P1042S Child/Adolescent and Parent/Caregiver Questionnaires and adaptation of the Diabetes Family Responsibility Questionnaire	The most frequently reported barrier by either group was forgetting to take medication. Logistical barriers including scheduling issues were more likely to be reported by children who knew their HIV status. Having a biological parent as a caregiver was associated with regimen and fear of disclosure being barriers.
Byrne et al. [14]	Longitudinal study that interviewed the primary caregiver and information was taken from patient’s chart.	Family structure, caregiver education, opinions concerning benefits of medicine, severity of child’s illness, child’s susceptibility to being sicker, barriers to taking specific medications, overall regimen, and strategies used by caregiver to ensure adherence	Estimate of doses missed in the past week, clinic appointments missed in the past 3 months, prescriptions unfilled in the past 3 months, and recall of medications prescribed; measured by visual analogue scale (VAS)	Interview	There was a high rate of adherence among participants. Issues related to adherence included stigma, disclosure issues, and family experiences. Caregivers and providers had different ratings for child adherence.
Childs and Cincotta [32]	Literature review: looks into describing a developmental approach that includes a comprehensive assessment to address multiple challenges faced by individuals and families.	Caregiver anxiety about medication, caregiver disclosure of child HIV status to school, school social dynamics, and child’s understanding of illness, parent HIV status	Parent self-report	Interview with social worker	Not disclosing HIV status to the school can lead to missing medication doses. The child’s lack of HIV knowledge can also lessen medication adherence. Not disclosing HIV status can also lead to feelings of secrecy and shame, therefore lowering adherence. Medication adherence can be difficult for a child if their parent has HIV and is not adherent to their medications and may ignore the child’s medication so that they don’t confront their illness. Another factor that may impact a child’s adherence is if their parent is in the fail stages of the disease and cannot physically provide or take care of their HIV + child. Parents without HIV may question the efficacy of the medication and may see negative side effects as a reason to not be compliant with the child’s medication. There may also be a misunderstanding of the child’s complex medication regimen that may cause parents to forget to provide the medication to their child.
Cunningham et al. [19]	Multisystemic therapy (MST) encompasses the individual adolescent, the family system, and the border community system and provides therapeutic services to change problem behaviors (i.e. medication adherence).	Goal of therapy: increase authoritative parenting, improve the bond between father and son, and improve the father’s medication-monitoring practices	Weekly checks, pill counts, and viral load	Children’s Depression Inventory	After the intervention, viral load decreased and remained undetectable for about a year. After the participant’s viral load increased again, the intervention was implemented a second time, causing viral load to decline in about 3 months.
Davey et al. [21]	Retrospective chart review	Family support	CD4 count, viral load, and whether or not medication was started within 6 months of diagnosis	Family Support Tool	Both before and after diagnosis, family support was generally mixed or unsupportive. 31% of participants started medication within 6 months of their diagnosis. Lack of family support for HIV + youth may contribute to the acquisition of HIV and further risk of poor adherence to HIV treatment.
Dodds et al. [33]	Descriptive analysis	Exposure to traumatic life events during childhood, number of children cared for, education level, and marital status	CD4 count	Mental health screening questionnaire	The women often didn’t keep up with their medical care due to mental health issues. They were less likely to take their medication when it caused interference with their social life. Lack of family or spousal support or fear of others finding out about the female’s HIV status may hinder the individual’s medication adherence or going to support services. Also, negative perceptions of HIV (such as an early death, or being a burden to their families) may reduce medication adherence.
Dolezal *etl al.* [26]	Cross-sectional	Caregiver HIV status, caregiver relationship to child, and child’s knowledge of HIV status	CD4 + count and percent, HIV RNA viral load, caregiver self-report of medication taken, child self-report of medication taken	Interview	There was little agreement between caregiver- and child-reported adherence.
Ellis et al. [22]	Multisystemic therapy (MST) home-based intervention	Aimed at improving adherence through family meetings where they worked on issues such as caregiver HIV knowledge	Viral load and caregiver-reported medication adherence	Caregiver questionnaire	The intervention improved caregiver HIV knowledge. Viral load decreased after the intervention. However, there was no change in caregiver-reported medication adherence.
Fair et al. [15]	Social work intervention consisting of home visits	Offering education about HIV and medication adherence to families	CD4 count and viral load	Home-based services	The intervention showed improved CD4 count and viral load among the participants.
Fields et al. [34]	Qualitative analysis of interviews with hopes of addressing problems and creating a customizable intervention by addressing barriers and their psychosocial antecedents.	Youth-reported barriers to ART adherence such as reactance, complicated regimens, HIV fatigue, transitioning to autonomous care	Percentage of doses taken in the last month	Center for Epidemiologic Studies Depression Scale and semi-structured qualitative interview	Youth who were perinatally infected with HIV reported barriers such as reactance, complicated regimens, HIV fatigue, and difficulty transitioning to autonomous care. Youth who were behaviorally infected with HIV reported barriers such as HIV-related shame and difficulty initiating medication. Both groups reported barriers such as substance use, medication side effects, unstructured lifestyles, depressed mood, poor risk perception, medication being a reminder of their HIV status, and a desire for privacy. 67% of the behaviorally infected youth were on medication, and 50% of them had adherence less than 85%. 83% of the perinatally infected youth were on medication, and 40% of them had adherence less than 85%. Part of the poor adherence stemmed from having negative family experiences (either a parent with HIV dying or poor family interactions) in which taking the HIV medication reminded the child of their dead parent, so taking the medication became less important. In terms of being the only child with HIV + status, many children did not take their medication because of feeling isolated, and many family members would remind the child of their “abnormality”, thus fueling the child’s sense of shame and lack of adherence.
Gray et al. [35]	Behavioral Family Systems Therapy (BFST) intervention consisting of weekly meetings with parent/adolescent dyads in an alternating home-based and telehealth format	Discuss current barriers to adherence and how this can be improved	Viral load, self-reported medication use, physician-estimated adherence, electronic medication use monitoring through Medication Event Monitoring System (MEMS) TrackCaps, and pill count	Pediatric AIDS Clinical Trials Group (PACTG) Adherence Module 2 and Client Satisfaction Questionnaire (CSQ)	Out of 4 participants, 3 had improved adherence, 2 had decreased viral load, and all had fewer barriers to adherence after the intervention. Working with caregivers and adolescents to address and generate effective solutions, helped with increasing the child’s adherence. By having both the caregiver and child come up with solutions, the adolescent was more likely to adhere to their medications. Successful solutions were finding ways to incorporate the regimen into the adolescents’ routine rather than having the adolescents go out of their normal routine to take their medication. However, any changes in the adolescent’s routine were found to decrease adherence.
Kacanek et al. [16]	Longitudinal study at 15 US clinical sites	Socioeconomic status, caregiver education, caregiver marital status, number of caregiver functional limitations, number of stressful events, and other social and structural factors	CD4% and ART regimen	Caregiver and child interviews	Participants aged 15–17 nonadherence was more likely to be associated with structural factors, such as exposure to violence, family factors, and desire for social acceptance such as through norm conformity. For younger participants utilizing a buddy system was associated with worse adherence. Knowing HIV status was associated with suppressed viral load. For all participants, nonadherence was associated with side effects and distressing physical symptoms.
Katko et al. [27]	Cross-sectional	Caregiver’s ability to verbalize medications	The proportion of days of medication dispensed to days of medication prescribed, adherence to appointments, and virologic response	Interview, review of pharmacy report of prescribed medications	Caregivers who could not describe their child’s medication regimen or did not adhere to appointments were less likely to demonstrate medication adherence.
Letourneau et al. [20]	Multisystemic Therapy (MST) intervention with participants and parents	Targeting “Fit factors” maintaining nonadherence including sexual risk and substance use behaviors, and medication nonadherence	Viral load, CD4 count, and pill counts	Assessment of fit interview	The participant’s nonadherence was associated with family-level factors such as low caregiver supervision/medication monitoring and permissive parenting style. The intervention led to improved medication adherence.
Lyon et al. [28]	FACE Advance Care Planning intervention which sought to promote conversations about end-of-life care.	Children and caregivers identified the most common reasons given for missing doses, followed by treatment groups aimed at addressing these issues	Self-reported adherence measured by the National Institute of Health Adherence to Medication Questionnaire, case manager-reported adherence, viral load, and CD4 count	CAPS interview of reasons given for missing doses	After the intervention, 91% of youth self-reported improvement in adherence. Four youth’s viral loads were reduced to undetectable, and another four youth’s CD4 counts improved. Family and treatment buddies said the program was highly helpful.
Malee et al. [36]	Cross-sectional	Conduct problems, learning problems, psychosomatic symptoms, impulsivity-hyperactivity, anxiety, general hyperactivity, and psychosocial factors, all rated by parents	Self-reported adherence measured based on ACTG instrument, viral load, and CD4 count	Conners’ Parent Rating Scale (CPRS- 48) and General Health Assessment for Children (GHAC)	Nonadherence was associated with behavioral impairment in at least one area (*p* =.04). Specifically, conduct problems (*p* =.005) and general hyperactivity (*p* =.02) were associated with nonadherence. Nonadherence was also associated with recent life stress (*p* =.01). Greater adherence was associated with an adult being responsible for administering medication.
Marhefka et al. [29]	Cross-sectional	Caregiver understanding and knowledge of prescribed regimen and caregiver-perceived barriers to adherence	Typical Dosing Interval and Interval Deviance, pharmacy refill rates, and viral load	Treatment Interview Protocol (TIP) - caregiver-completed structured interview	Being able to identify prescribed medications was associated with a higher pharmacy refill rate (*p* <.01). There were 21 barriers to adherence identified by caregivers. Identifying more barriers was associated with worse adherence (*p* <.05).
Marhefka et al. [46]	Cross-sectional, descriptive	Parenting stress, psychological distress, perceived helpfulness of available social support, and perceived need for social support	Pharmacy refill records and viral suppression rates	Parenting Stress Index (PSI) Short Form - Second Edition, Brief Symptom Inventory (BSI), Family Support Scale (FSS), and Support Functions Scale (SFS)	Adherence did not have a significant association with parenting stress, perceived helpfulness of available social support, or perceived need for social support. Adherence was associated with lower psychiatric distress among caregivers (*p* =.005).
Martin et al. [17]	Longitudinal study	Responsibility for medication-related tasks, knowledge of HIV and adherence, regimen knowledge, and regimen complexity	Electronically-monitored adherence measured by Medication Event Monitoring System (MEMS) Track caps, viral load, and CD4 levels	Responsibility for Medication Scale (RMS), general knowledge of HIV subscale (K-HIV), 6-item subscale assessing knowledge about adherence (K-ADH), and Pills Identification Test (PIT)	8% of child-caregiver dyads agreed on who was responsible for tasks regarding medication. Greater agreement about these responsibilities, along with greater caregiver regimen knowledge, were associated with better adherence.
Mellins et al. [37]	cross-sectional	Disclosure of HIV status to child, caregiver, and child mental health, and parent-child relationship	CD4 count and viral load	Semi-structured interviews, Beck Depression Inventory (BDI), Child Behavior Checklist - Parent Version (CBCL), Child Depression Inventory (CDI), State-Trait Anxiety Inventory - Youth Form (STAI), and Parent-Child Relationship Inventory (PCRI)	Greater social disclosure was associated with a lower CD4 count, negative HIV status for the caregiver, and the caregiver not being the child’s biological parent.
Mellins et al. [30]	Cross-sectional with psychological assessments and self-report adherence data collected from caregivers	Child level of responsibility for taking medications, child and caregiver cognitive functioning, child and caregiver mental health, child stressful life events, caregiver perceived stress, caregiver-child relationship, caregiver quality of life, caregiver self-efficacy to adhere to child’s HIV treatment, and social disclosure	Child- and caregiver-reported adherence measured by modified Adult AIDS Clinical Trials Group (AACTG) procedures, HIV-related symptoms, and medication burden	Trail Making Test, Child Behavior Checklist - parent version, Child Depression Inventory, Perceived Stress Scale, Parent-Child Relationship Inventory, Medical Outcomes Survey - HIV, and Adherence Self-Efficacy Scale	Worse parent-child communication (*p* <.017), higher caregiver stress (*p* <.002), lower caregiver quality of life (*p* <.003), and worse caregiver cognitive functioning (*p* =.033) were significantly associated with nonadherence.
Naar-King et al. [31]	Cross-sectional	Caregiver health status, family cohesion, expressiveness and conflict, caregiver perceived support, stressful life events, and relationship with provider	Caregiver-reported adherence measured by a modification of the Diabetic Self-Care Practice Instrument and viral load assays	Health Status Questionnaire, Family Relationship Index (FRI), Personal Resource Questionnaire (PRQ), Family Inventory of Life Events (FILE), and Parent Perceptions of Specialty Care (PPSC)	Family factors (family cohesion, expressiveness, and conflict) had no significant association with adherence.
Naar-King et al. [38]	Cross-sectional with descriptive analysis	Allocation of family responsibility	Viral load and youth and parent self-reported adherence	Adaptation of the Diabetes Family Responsibility Questionnaire (DFRQ)	There was an association between higher caregiver responsibility for medication and higher adherence (*p* =.004).
Nicholson et al. [44]	Cross-sectional study with semi-structured interviews with caregivers	Caregiver knowledge of HIV, education level, socioeconomic status, and number of caregivers	Caregiver self-report measured by ACTG adherence scale, pill counts, HIV RNA viral load, and CD4 lymphocytes subset number and percent	True-false assessment, AIDS Clinical Trials Group (ACTG) Adherence Self-Efficacy Scale, and Trail Making Test	Lower adherence was associated with caregiver belief that it interfered with their child’s social status. Caregiver self-efficacy was correlated with higher child CD4 + counts.
Rao et al. [39]	Focus group intervention: adolescents to explore attitudes and experiences around medication adherence	Stigma from family members and status disclosure	Self-reported adherence	Questions facilitated by a focus group leader	50% of participants reported skipping doses out of fear of family discovering their HIV status. 64% agreed that medication side effects do not bother them.
Reddington et al. [18]	Longitudinal medical record review study	Family structure, daily routine changes, and caregivers’ attitudes toward medication administration	Viral load	Interview	The more drugs participants were taking, the more challenging adherence was. Caregivers of nonadherent children stated needing more emotional support and help at home.
Reisner et al. [24]	Literature review that included qualitative and quantitative studies on HAART, young adults, and medical adherence (or lack thereof)	Socioeconomic indicators, education level, who the family/caregiver is and their education level, social support, and mental health status	Self-report, others’ report, pill count, pharmacy refill records, plasma HIV RNA level, and CD4 count	Compilation of studies	Five types of factors were found to be associated with the level of adherence. They are demographic factors, psychosocial factors, HIV disease factors, treatment regimen factors, and practitioner factors. Some of the many factors found to be associated with nonadherence were unstable housing, HIV stigma, and discrimination by friends and family. Factors associated with adherence included an adult other than a biological parent being a primary caregiver and a higher caregiver education level.
Simoni et al. [45]	Qualitative systematic review	Resources and efficacy of caregivers, parental illness and comorbidities, parental guilt about infection, who administers medication, accuracy of information caregiver has, parent-child communication, caregiver stress, caregiver quality of life, caregiver cognitive functioning, and parental perceived vulnerability and barriers	Biological assays, self-report, caregiver reports, clinician assessments, medical chart reviews, clinic attendance, pill count, pharmacy refill records, electronic drug monitoring (EDM), directly observed therapy (DOT), resistance testing, viral load, and CD4 count, CDC-defined stage of disease progression, and mortality	Caregiver questioning	Based on all articles included in the review, it was found that family and caregiver factors have a large impact on medication adherence due to youth’s dependency on their caregivers to receive medication. Caregivers play a crucial role in whether or not youth adhere to HIV treatment.
Steele and Grauer [40]	Literature review	Environmental difficulties, child behavior, housing instability, living situation, caregiver beliefs, social support, and negative life events	Blood assay, parent report, clinician estimate, self-report, pharmacy records, pill count, chart review, electronic data, immunosuppression, estimated adherence via Visual Analog Scale (VAS), adherence as determined by the Pediatric AIDS Clinical Trials Group (PACTG) adherence modules, and Medication Event Monitoring System caps (MEMS caps)	Caregiver questioning	Based on all articles included in the review, children and adolescents’ medication adherence is often lower than recommended. Nonadherence is connected to a variety of parental/caregiver factors such as medication issues, cognitive/emotional reasons, scheduling problems, housing instability, length of treatment, depressive symptoms, parental belief in adherence inefficacy, greater caregiver concern about the child’s HIV status being discovered, and negative life events.
Steele et al. [40]	Cohort survey	Caregiver-reported perceived vulnerability and caregiver-reported difficulty resulting from barriers	Caregiver-reported adherence, pill count, and APREX TrackCaps data	Survey and semi-structured interview	There was no significant relationship between medication adherence and perceived vulnerability and barriers.
Thrul et al. [41]	Qualtrics survey to healthcare providers and retrospective chart review of patients	Provider viral suppression predictions and reasons for/factors contributing to these predictions	Viral load	Qualtrics survey	The providers’ viral suppression predictions had low accuracy (59%). Reasons cited for low viral suppression predictions included medication nonadherence, new patient status, and vulnerabilities such as unstable housing. Reasons cited for predictions of viral suppression included medication adherence, history of viral suppression, and the presence of family and other social support.
Williams et al. [42]	Cross-sectional study	Stressful life events, caregiver education, and family structure	CD4 level, viral load, and self-reported adherence	Questionnaire	Family factors such as recent stressful life events (*p* =.01), repeating a grade in school (*p* =.004), and depression or anxiety diagnosis (*p* =.07) were associated with non-adherence. Factors associated with increased adherence were the primary caregiver being a non-biological parent (*p* <.001), usage of a buddy system strategy for remembering medication (*p* =.05), and higher caregiver education level (*p* =.001).
Wrubel et al. [43]	Qualitative analysis: narrative responses from mothers in interview	Mothers’ attitudes and feelings related to adherence practices, interactions of mothers and children related to adherence practices, mothers’ general appraisal of HIV medication, belief in medication efficacy, and feelings of guilt	Commitment to adherence and qualitative self-reported adherence	Qualitative interview	Mothers’ commitment to adherence had a positive effect on adherence, whereas feelings of stigma and guilt, effects of bereavement, and children adopting mother’s attitudes towards medication had a negative effect. Factors important to the process of administering medication were child behavior, mothers’ developmental expectations, and mothers’ adherence to their own HIV medication.

One study was a home-based nursing intervention in which nurses completed 8 structured home visits over three months and provided education about participants’ medications and the HIV disease to both children and parents. They also worked with families to provide strategies and tools for improving adherence such as medication boxes, pill crushers, pill-swallowing training, and toys as reward incentives. The goal of these visits was to improve adherence through both education and addressing barriers to adherence. For the control group, education was provided, and adherence strategies were offered in the clinic, rather than the home, and appointments only took place about once every three months. In the event of nonadherence, phone calls were made to patients’ homes, and home visits were arranged if no other strategies were successful [23].

The first MST intervention highlighted a single case in which the main goals were to increase the father’s authoritative parenting, improve the father and son’s bond, and improve the father’s practices of monitoring medication. The strategies implemented by the therapist included role-playing and teaching communication skills. The therapist also provided the patient with strategies for mitigating depressive symptoms and for swallowing large pills [19]. In the second MST intervention, therapists met with families two to three times a week. Therapists provided services such as communication skills training, introduction of discipline systems, and helping families keep appointments, depending on what each family needed [22]. The third MST intervention was another case study focusing on one patient. This patient’s specific barriers being addressed included medication nonadherence, sexual risk, and substance use. The intervention consisted of education on medication effectiveness and the consequences of the patient’s behaviors. The therapist also provided parental education on appropriate discipline in such situations [20].

One study consisted of a social work-based home intervention. The goal of the intervention was to improve medication adherence and access to services. It consisted of daily phone-based communication between the social worker and the patient, offering HIV education to parents, and home visits with rewards for improved lab results [15]. Another study focused on intervening by implementing Behavioral Family Systems Therapy (BFST). The intervention was carried out in seven weekly dyad meetings consisting of HIV education, problem-solving, and family communication skills training, and highlighting family roles and expectations [35].

The focus group study implemented preliminary focus groups aimed at having youth identify barriers to adherence. Caregivers ranked these barriers in order of importance, and feedback was obtained on which interventions were thought to be most helpful. Based on this, groups of 16–20 youths and caregivers were conducted targeting these issues. Group sessions consisted of caregiver and youth education and youth group discussions [28].

### Study Design, Outcome, and Participant Eligibility

The study containing the home-based nursing intervention was a randomized controlled trial whose primary outcome measures were self-reported adherence determined by a questionnaire and pharmacy refill records, based on how often refills were being obtained. Participants were HIV-positive patients aged 1.5–20 years who were enrolled in a pediatric HIV program [23]. Of the MST interventions, two were case studies focusing on one patient, and one was a retrospective chart review. For one of the case studies, the main outcome measure was having an improved viral load measure. The participant was included in the study after being referred for a high viral load (> 45,000) [19]. The other case study’s primary outcome measures for adherence were pill counts, reduced and/or undetectable viral load, and CD4 count > 200, 200 or less being an indicator of severe immune deficiency. The participant was recruited due to being perinatally infected with HIV and displaying multiple HIV risk behaviors [20]. The retrospective chart review assessed improved viral load and caregiver questionnaire responses as outcome measures and included participants between 20 months and 16 years of age who were perinatally infected with HIV. Participants were referred to the program if their viral load was 5000 HIV RNA copies per milliliter (c/ml) or higher, and their self or caregiver-reported adherence was less than 80% [22]. The social work home intervention utilized a longitudinal mixed-methods design with outcome measures of increased CD4 count and decreased or undetectable viral load. Participants in this study were HIV-positive patients between 10 and 21 years of age who were receiving treatment and identified as at risk due to their high viral loads [15]. The BFST intervention was a case series intervention in which outcomes were decreased viral load as assessed from medical records, medication use monitored from Medication Event Monitoring System (MEMS) TrackCaps, pill counts calculated as number of pills taken divided by number of pills expected to be taken, self-reported adherence based on a paper log, and physician-estimated adherence by visual analog scale. Participants were children aged 13–17 years who were aware of their HIV diagnosis and had less than 80% adherence to HAART [35]. The focus group study was a pilot intervention project. The project’s outcomes were self-reported adherence of at least 90% based on the National Institute of Health Adherence to Medication Questionnaire and the CAPS interview of reasons given for missing doses, at least 90% adherence based on case manager report, CD4 count above 500, and one-log reduction in viral load in addition to maintaining an undetectable viral load. The adolescents, aged 15–22 years, and their family members were recruited from a clinic based on case manager-reported struggles with adherence [28].

### Intervention Effectiveness

The home-based nursing intervention showed improved, however, not statistically significant, self-reported adherence in the intervention group compared to the control group (*p =*.07). The mean pharmacy refill score, on a scale of 0–3, for the intervention group was 2.7, and 1.7 for the control group (*p =*.002) [23]. The MST case studies both saw an improvement in adherence. The first participant’s viral load went from a monthly average of > 20,000 to < 200 in the first month, followed by undetectable levels soon after. After leaving the program and experiencing a viral load increase to a monthly average of > 13,000, the participant re-enrolled in the program and had a viral load decline of 1 log by the end of the third month of treatment [19]. The other case study saw the participant’s viral load decrease from an average of 12,788 copies/ml before treatment to nondetectable levels. Additionally, pill counts showed 70–80% adherence after 4 weeks and 93–100% in the last 3 months. The participant’s CD4 count was similar (> 400) before and after the intervention [20]. The MST retrospective chart review, despite improving caregiver HIV knowledge, showed no significant effect on caregiver-reported adherence, likely since adherence was reported as 98%, on average, at entry. There was an average viral load decrease of 1.3 log_10_ among participants, and the change in viral load from before to after treatment was statistically significant (t = 5.41, *p <*.001) [22]. The social work home intervention saw a significant increase in CD4 counts [t(14) = − 2.35, *p =*.03] and a significant decrease in viral load [t(14) = 2.08, *p =*.05] [15]. The BFST intervention reported a combined adherence percentage, a physician-estimated adherence percentage, and viral load data. Of the 4 participants, 3 of them had an improved adherence, all had increased physician-estimated adherence, and 2 had decreased viral load [19]. As a result of the focus group pilot project, 91% of participants reported improved adherence, which was confirmed by their case managers. 43.5% of participants had improved CD4 counts (above 500). Four participants’ viral loads decreased to undetectable levels during the treatment [28].

### Non-Intervention Studies

Besides intervention studies, there were *n* = 29 studies remained which examined family factors in relation to adherence. When looking at how the studies measured family factors, responses came from three different groups: caregiver and child (*n* = 12), the caregiver (*n* = 10), the youth (*n* = 5), and a medical professional (*n* = 2). Common measurements were qualitative interviews (*n* = 17), questionnaires (*n* = 10), retrospective chart review (*n* = 1), and various unknown measurements (*n* = 1).

### Family-Level Factors

The 29 studies measured four major themes of potential familial influences on medication adherence: knowledge of medication regimen by the parent and/or child, social relationships, the general health of the parent and/or child, and general barriers self-reported by either the caregiver or the child.

### Knowledge

A total of *n* = 12 studies fell into this category. A total of *n* = 6 studies measured the caregiver and/or child’s perceived difficulty in administering dosages, *n* = 2 studies examined the participant’s HIV knowledge, *n* = 3 studies asked caregivers the extent to which their child understands their HIV status, and *n* = 3 studies asked caregivers to identify the medications and/or dosages of the HIV + child.

### Social Relationships

A total of *n* = 14 studies investigated social relationships and their correlation with HIV medicine adherence. Many of these studies (*n* = 5), examined the child-caregiver relationship, followed by the child’s transparency to their parent about taking their medication (*n* = 4), general family support (*n* = 5), school social dynamics (*n* = 3), and allocation of responsibility the parent gives their child with their medication regimen (*n* = 2).

### Health

A total of *n* = 12 studies researched the impact of parental and/or child health on the child’s adherence to their medication. The most prominent factor studied within this category was trauma or any stressful events inflicted upon the child and/or parent (*n* = 7), followed by mental health status (*n* = 4), parent HIV status (*n* = 2), and any functional disabilities (*n* = 1).

### General

A total of *n* = 8 studies inspected any additional barriers to adherence and compiled a list of environmental and youth risk factors.

#### Outcome Measurements

Three major methods were used to measure adherence: pill counts, CD4 counts, and HIV RNA viral loads. Several studies (*n =* 11) compiled information on pill counts from pharmaceutical records or self-reports from participants, followed by the combination of all three methods (*n* = 7), only viral load and pill counts (*n* = 4), HIV RNA viral loads (*n* = 2), CD4 counts (*n* = 2), CD4 and pill count (*n* = 1), viral load and CD4 counts (*n* = 1), and only self-reports from participants (*n* = 1).

### Key Findings

Having a caregiver in charge of administering medication to the child generally resulted in greater adherence (*p =*.004) and was associated with a lower CD4 count, especially if the child’s biological parent was not the primary caregiver [24, 28, 36–38]. In contrast, lower caregiver knowledge (*p =*.01) and difficulty in administering the medication (*p =*.03) were associated with higher viral load [12, 27]. Many studies found that complicated regimens presented a challenge to many HIV-infected youth who found themselves overwhelmed by keeping track of their prescriptions and remembering to take their medication promptly [16, 25, 29, 34, 39–41]. The more drugs the HIV-infected youth took, the more challenging it was to adhere to the schedule [18].

In addition, those who were behaviorally infected found it difficult to initiate their medication because of HIV-related shame and found difficulty in transitioning to adult care due to a lack of social support [34, 40]. The presence of a support system was identified as vital in ensuring adherence across studies. Issues such as stigma associated with HIV and negative family experiences were also commonly correlated with lower adherence, as the child is less likely to disclose their HIV status, and therefore, skip their medications to avoid feeling alienated by their peers and family members [23, 33, 39, 40, 42, 43].

In several studies, participants were less likely to take their medication when it interfered with their social life. Not disclosing their HIV status to their peers or family members not only correlated with missed doses but was also associated with a higher CD4 count [16, 33, 37]. Davey et al. [21] illustrated this point in which they measured how supportive a child’s family was before and after receiving their HIV diagnosis. The consensus skewed neutral or unsupportive. Due to the overall lack of support, only 31% of participants started medication within 6 months of their diagnosis and had poor adherence to their HIV treatment [40]. As many as 50% of participants in one study skipped doses due to fear of their families discovering their HIV status [39]. Most youth across studies depended on their caregivers for medications and support. Any negative comments or reminders of the child’s condition fueled a sense of shame and lack of adherence [33, 34, 44, 45].

Living with parents without HIV, children with HIV may question the efficacy of the medication and may see the negative side effects as a reason for non-compliance [12, 32, 34]. Similarly, children with HIV + parents who do not take their medications or ignore the child’s medications may reinforce the practice of non-adherence, especially for younger children, who are more impressionable and have less HIV knowledge [32]. Conversely, caregivers with HIV, especially mothers, who adhere to their medications are more likely to have a positive effect on their child’s adherence and impart more HIV knowledge to their child, which is associated with a suppressed viral load [16, 17, 43].

Through parent-child questionnaires and interviews, studies found a lack of communication between the child and their caregiver was significantly associated with nonadherence [30, 45, 47]. In one study, only 8% of child-caregiver dyads agreed on who was responsible for administering medication [30]. This misalignment of answers was found to be another barrier to HIV adherence among families.

#### Barriers to Adherence

Non-adherence was also connected to a variety of health-related factors, specifically mental health issues and stressful events. In one study, participants described mental health symptoms when discussing adherence barriers they faced [37]. A higher adherencewas correlated with lower psychiatric distress among caregivers (*p =*.005), while depression and anxiety increased non-adherence (*p =*.07) as measured by lower CD4 counts and pharmacy refills [33, 40, 42, 46]. General behavioral impairments among children, especially conduct problems (*p =*.005) and general hyperactivity (*p =*.02) were associated with higher viral load and lower adherence [36]. Dealing with a child with behavioral issues was correlated with higher caregiver stress (*p <*.002), lower caregiver quality of life (*p <*.003), and worse caregiver cognitive functioning (*p =*.033), which further influenced adherence [30]. Similarly, recent stressful events on both the child and parent significantly impacted non-adherence (*p =*.01) [36, 40, 42].

#### Environmental Factors

Beyond health factors, many healthcare providers and researchers find access to resources is vital to overcome adherence barriers. Many families have self-reported that unstable housing, economic difficulties, and scheduling problems have been reasons for low adherence, with one or more of these barriers with even less adherence (*p <*.05) [24, 29, 41, 44, 47].

#### Family Dynamics as Moderators/Mediators

No family-level moderators or mediators were identified in the studies reviewed. Although the studies extensively examined various family-related factors such as caregiver knowledge, family dynamics, and parental health, no research explicitly focused on the role of family-level moderators or mediators that could influence the relationship between these factors and adherence outcomes.

## Discussion

In this systematic review, we aimed to synthesize the current body of literature on family interventions and factors influencing ART adherence among children, adolescents, and young people with HIV in the United States. The findings highlight several key themes, including the critical role of family involvement on adherence outcomes in this population. Specifically, in this review we sought to answer three research questions: First, what is the impact of family-based interventions on ART adherence in youth with HIV in the United States? Second, what family-level factors influence ART adherence in this population? Third, how do family dynamics mediate or moderate ART adherence behaviors in youth with HIV? The findings address each of these questions, with evidence supporting the importance of family involvement, mental health, and social determinants of health as key components in influencing ART adherence.

One of the most consistent findings across the studies was the importance of caregiver involvement in the administration of ART. Studies demonstrated that when caregivers were responsible for medication management, adherence rates were significantly higher as indicated by reduced viral loads and improved CD4 counts. This finding aligns with previous research that has emphasized the role of family support in managing chronic illnesses in pediatric populations. As hypothesized in our first research question, family-based interventions that involve caregiver support and involvement, such as those using multisystemic therapy (MST) and Behavioral Family Systems Therapy (BFST), can significantly improve adherence outcomes [12, 19, 20, 28]. However, when caregivers had limited knowledge of the child’s medication regimen or experienced difficulties in administering ART, adherence outcomes were poorer, which suggests a need for ongoing caregiver education and support to optimize treatment success, as well as the importance of family-level interventions to promote disclosure of HIV status and increase family support in these populations [24, 30].

The review also identified significant barriers to ART adherence, including complex medication regimens, HIV-related stigma, and psychosocial stressors. Adolescents, in particular, reported challenges in maintaining adherence due to the complexity of their ART schedules, which is consistent with prior research indicating that more complicated regimens pose a greater adherence burden. Moreover, HIV-related stigma emerged as a key barrier, with many youth expressing fear of disclosing their HIV status to peers or family members, which directly contributed to missed doses and higher viral loads [23, 33, 39]. This is particularly concerning for behaviorally infected youth, who not only face challenges in initiating ART but also struggle with the transition to autonomous care due to limited social support. As per our hypothesis in the second research question, we found that social factors like stigma and caregiver support were critical influences on adherence behavior [34, 39, 44]. These findings further reinforce the potential need for family level interventions to strengthen family relationships in this population.

Interventions targeting family dynamics, such as MST and BFST, showed promise in improving adherence outcomes. These interventions provided families with the tools needed to address both practical barriers to adherence, such as medication management, and psychosocial issues, such as family communication and mental health challenges. In particular, MST interventions demonstrated substantial improvements in viral load reduction and medication adherence in case studies and retrospective reviews. These findings suggest that addressing the broader familial context, including communication patterns and caregiver support, is crucial for improving ART adherence. As anticipated in our third research question, family dynamics, including communication and emotional support, were found to mediate adherence behaviors [19, 20, 28].

While the importance of family factors and interventions were noted across studies, mental health and trauma concurrently played significant roles in ART adherence, with higher levels of psychiatric distress among both caregivers and children correlating with lower adherence rates. Depression, anxiety, and conduct problems among children were consistently linked to poorer adherence, underscoring the need for integrated care models that address both mental health and HIV treatment. Furthermore, stressful life events, including trauma and unstable living conditions, were significant predictors of non-adherence, highlighting the importance of addressing social determinants of health when designing interventions for HIV-positive youth. Family level interventions that target both family dynamics and psychopathology, such as Brief Strategic Family Therapy (BSFT), may be a promising approach for this population [48]. BSFT is particularly effective in addressing the intertwined challenges of family dynamics and mental health symptoms that impact adherence (externalizing and internalizing symptoms). This therapeutic model emphasizes restructuring family interactions, improving communication, and fostering healthier family relationships, while also integrating strategies to manage and reduce psychiatric distress. By focusing on the interaction patterns that perpetuate maladaptive behaviors, BSFT helps families identify and modify unhelpful dynamics that may be exacerbating mental health symptoms or non-adherence to treatment. BSFT has also been tested among minoritized populations, who were the majority of participants across the studies. Given the multifaceted challenges HIV-positive youth face, particularly those related to family dynamics, trauma, and mental health, incorporating BSFT into care models provides a holistic approach that not only targets the individual but also strengthens the family system, creating an environment more conducive to sustained ART adherence [9, 12, 20].

Similarly, while family dynamics were central to ART adherence, environmental factors such as economic difficulties, housing instability, and access to healthcare also played significant roles within the family context. Families experiencing these structural challenges often struggled with ART adherence, which in turn impacted the health outcomes of children and adolescents with HIV. Just as mental health issues require integrated care models, interventions that address the broader social determinants of health—particularly those affecting family stability—may be crucial. Family-centered interventions that not only focus on communication and support but also help families navigate and access critical resources, such as stable housing and financial support, may hold promise in promoting sustained adherence. Addressing these environmental barriers is essential for ensuring that families are equipped to provide the support needed for optimal ART adherence in HIV-positive youth [7, 8, 35].

### Implications for Practice and Policy

The findings of this review provide compelling evidence for the critical role of family-centered interventions in improving ART adherence among children, adolescents, and young people living with HIV. Healthcare providers and policymakers should focus on developing and implementing multilevel interventions that holistically address the various factors influencing adherence, with families at the center of these efforts. Several key implications for practice and policy emerge from this review.

First, enhancing caregiver involvement in ART administration is essential. Interventions should focus on providing caregivers with the knowledge and tools necessary to effectively manage their child’s medication regimen. This includes education about HIV and ART, training in medication administration, and support for maintaining adherence. Family-based interventions should also aim to foster open communication about HIV status within the family, as disclosure and support are critical for adherence, particularly among adolescents. Programs such as MST and BFST have shown promise in addressing these needs, offering a template for future interventions [19, 20, 28].

Second, integrating mental health services into HIV care is crucial for addressing the psychiatric distress that often accompanies HIV in both caregivers and youth. Mental health disorders such as depression, anxiety, and conduct problems are significant barriers to ART adherence, and untreated psychiatric symptoms can further strain family dynamics. Interventions that address both mental health and ART adherence, such as Brief Strategic Family Therapy, may be particularly effective for this population. Policymakers should consider funding and supporting integrated care models that combine HIV treatment with mental health services, recognizing the critical role of mental health in treatment outcomes [9, 16, 48].

Third, addressing the social determinants of health that impact ART adherence is essential for ensuring that family-centered interventions are effective. Structural challenges, such as housing instability, economic hardship, and limited access to healthcare, were frequently cited as barriers to adherence in this review. Interventions must therefore go beyond the immediate family unit to include strategies that help families overcome these broader barriers. This may involve policies that provide financial support, housing assistance, and expanded healthcare access to low-income families affected by HIV. Additionally, healthcare providers should be equipped to connect families with community resources that can alleviate these burdens [7, 8, 35].

Fourth, developing policies that support long-term family stability is key to improving adherence outcomes. This includes advocating for systemic changes that reduce economic disparities and improve access to social services for families affected by HIV. Housing programs that provide stable environments for families, along with employment and income support, are necessary to help alleviate the financial strain that often accompanies chronic illness management. Addressing these structural issues will empower families to provide the support their children need to maintain consistent ART adherence [7, 8, 35].

Lastly, creating tailored interventions for behaviorally infected youth is an important consideration. This group faces unique challenges related to stigma, disclosure, and transitioning to autonomous care, and family interventions should be designed with these specific issues in mind. Policies that support the development of specialized interventions for behaviorally infected youth, while also strengthening the family unit, will be critical in improving adherence outcomes in this population [34, 40, 41].

In conclusion, the evidence from this review suggests that a comprehensive approach is needed to support ART adherence in HIV-positive youth. By developing family-centered, integrated interventions that address both the psychosocial and structural factors impacting adherence, and by supporting these efforts with appropriate policies, healthcare providers and policymakers can improve health outcomes for this vulnerable population.

## Limitations

While this review provides important insights into the role of family factors and interventions in ART adherence among children and adolescents, several limitations must be acknowledged. First, the review was limited to studies conducted in the United States, which restricts the generalizability of the findings to other contexts, particularly in low- and middle-income countries where the HIV epidemic may present different challenges and healthcare systems. Second, the majority of the studies included in the review relied on self-reported measures of ART adherence, which are prone to social desirability and recall biases, potentially overestimating adherence levels. Objective measures, such as electronic monitoring systems or pharmacy refill records, were underutilized, which limits the accuracy of the adherence data. Third, there was significant heterogeneity in the types of family factors interventions reviewed, which made it difficult to directly compare outcomes across studies. The varied methodological approaches across the included studies also contributed to this challenge. Finally, although most studies included diverse samples, only a few studies considered the unique experiences of sexual and gender minority youth, indicating a gap in the research on how these intersecting identities might influence ART adherence.

### Future Research

Future research should aim to address these limitations by expanding the geographic scope of studies to include diverse global populations, particularly in resource-limited settings. Additionally, greater emphasis should be placed on utilizing objective adherence measures, such as Medication Event Monitoring Systems (MEMS) or electronic pill trackers, to obtain more accurate assessments of ART adherence. Longitudinal research is also needed to understand the sustained impact of family interventions over time, especially as children transition into adolescence and eventually autonomous care. Moreover, future studies should explore how intersecting identities, including sexual orientation, gender identity, and racial or ethnic background, influence ART adherence and the effectiveness of family interventions. There is also a need for research on innovative intervention strategies that integrate technology, such as mHealth tools, to provide continuous support for both youth and their families. Lastly, exploring the role of mental health services and trauma-informed care in conjunction with ART adherence interventions is essential, given the strong associations between psychosocial stressors and non-adherence identified in this review.

## Conclusion

This review highlights the critical role of family dynamics in influencing ART adherence among children, adolescents, and young people living with HIV in the United States. Family-centered interventions, particularly those that address both practical and psychosocial barriers to adherence, demonstrate promise in improving health outcomes. However, adherence is a complex and multifaceted issue, influenced not only by family support but also by mental health, stigma, and environmental challenges. As such, successful interventions must take a holistic approach, addressing both medical and psychosocial factors to ensure long-term adherence. Future research should continue to explore these dynamics, with a focus on objective adherence measures, diverse populations, and the integration of mental health and trauma-informed care into adherence interventions. By doing so, the field can move toward more effective, equitable, and sustainable solutions for youth living with HIV.
